# Buzz Factor or Innovation Potential: What Explains Cryptocurrencies’ Returns?

**DOI:** 10.1371/journal.pone.0169556

**Published:** 2017-01-13

**Authors:** Sha Wang, Jean-Philippe Vergne

**Affiliations:** 1 Economics Department, Western University, London, Ontario, Canada; 2 Ivey Business School, Western University, London, Ontario, Canada; University of Rijeka, CROATIA

## Abstract

Cryptocurrencies have become increasingly popular since the introduction of bitcoin in 2009. In this paper, we identify factors associated with variations in cryptocurrencies’ market values. In the past, researchers argued that the “buzz” surrounding cryptocurrencies in online media explained their price variations. But this observation obfuscates the notion that cryptocurrencies, unlike fiat currencies, are technologies entailing a true innovation potential. By using, for the first time, a unique measure of innovation potential, we find that the latter is in fact the most important factor associated with increases in cryptocurrency returns. By contrast, we find that the buzz surrounding cryptocurrencies is negatively associated with returns after controlling for a variety of factors, such as supply growth and liquidity. Also interesting is our finding that a cryptocurrency’s association with fraudulent activity is not negatively associated with weekly returns—a result that further qualifies the media’s influence on cryptocurrencies. Finally, we find that an increase in supply is positively associated with weekly returns. Taken together, our findings show that cryptocurrencies do not behave like traditional currencies or commodities—unlike what most prior research has assumed—and depict an industry that is much more mature, and much less speculative, than has been implied by previous accounts.

## Introduction

Since the introduction of bitcoin in 2009 [1], cryptocurrencies have become increasingly popular. Cryptocurrencies are digital tokens that can be exchanged online, using cryptographic hashing and digital signatures to verify transactions and avoid double-spending of the same token. Thanks to these technical features, cryptocurrencies have introduced the notion of scarcity to the digital world by preventing users from copying the bytes that represent the token [2, 3]. Because the scarcity of cryptocurrencies is protected by the cryptography embedded in their open-source code (typically auditable by anybody), cryptocurrencies can potentially become valuable. But what explains fluctuations in their market value?

An obvious answer to this question is “supply and demand” [4, 5]. Typically, a cryptocurrency supply is predetermined by the underlying code, so market actors can anticipate most of the future variations in supply. For instance, 25 new bitcoin are generated every 10 minutes on average to reward those who lend computer power to verify transactions; this reward is halved every four years, until the supply of bitcoin reaches its programmed maximum of 21 million. Shifting patterns in mining activity (e.g., a mining rig’s bankruptcy) can affect the predictability of this average trend in the short term by slowing or accelerating the speed of transaction verification, but in the long run, the software protocol will adjust the network’s parameters to retarget the same average speed (in the case of bitcoin, 10 minutes between blocks). Thus, a cryptocurrency’s supply can vary in unexpected ways in the short term, but the long-term supply remains highly predictable by the ecosystem’s stakeholders. Since the bulk of variation in supply can be factored in user expectations, demand is the primary driver of cryptocurrencies’ fluctuations in value over time [6].

Many have warned against the “buzz factor” surrounding cryptocurrencies, which could cause large demand shocks in the short term [4, 6, 7]. For instance, bursts of media visibility [8] can attract waves of new users, and this movement can be partly anticipated by various market actors, such as cryptocurrency traders, thereby leading to price bubbles. For instance, a BBC writer speculated: “Has Bitcoin’s rising profile boosted its price? Michael Jackson, a partner at Mangrove Capital Partners says some changes in the price of Bitcoin have clearly been because of demand fuelled by media coverage” [9].

But the “buzz factor” hypothesis obfuscates the possibility that cryptocurrencies may also gain value and generate returns because they entail a true innovation potential. For instance, the technology underlying bitcoin enables fast international value transfers at very low fees (<1%) compared with the fees levied by banks and other payment-processing companies (e.g., Western Union can charge fees of up to 9%). Thus, cryptocurrencies are not only scarce but also potentially useful, which is likely to drive up their demand independently of short-term media cycles [10–11]. As explained by Ben Bernanke, then Chairman of the U.S. Federal Reserve: “innovations [such as bitcoin] may hold long-term promise, particularly if [they] promote a faster, more secure and more efficient payment system” [8]. But because prior research treated cryptocurrencies mostly as money rather than as technology, researchers never tested the relationship between innovation potential and cryptocurrency prices.

To remedy this shortcoming and better understand the factors explaining cryptocurrencies’ values, we model the evolution of weekly returns for five major cryptocurrencies over an entire year. Our findings show that the innovation potential embedded in technological upgrades is the most important factor associated (positively) with cryptocurrency returns. By contrast, we find that, after controlling for a variety of factors, such as supply growth and liquidity, the buzz surrounding cryptocurrencies is negatively associated with weekly returns. Also interesting is our finding that reports of fraudulent activity in the media are not significantly associated with returns—a result that further qualifies the media’s influence on cryptocurrencies.

Finally, we find that upward variations in supply are positively related to returns. This result warrants a detailed discussion at the end of this paper since it appears to be at odds with the Quantity Theory of Money [12], according to which, an increased supply should, ceteris paribus, lead to lower prices—and lower returns. This observation potentially implies that cryptocurrencies, at the microeconomic level of supply and demand, do not behave like traditional currencies, in contrast to the assumptions of most of prior research. Taken together, our findings emphasize the crucial role played by the technological upgrades underpinning innovation potential in the cryptocurrency ecosystem, and depict an industry that is much more mature, and much less speculative, than has been implied by previous accounts.

## Overview of Methodology

### Sampling Strategy

Since the launch of the first cryptocurrency, bitcoin, in 2009, dozens of other cryptocurrencies have been created. Most of them, though, do not represent serious attempts at establishing a foothold in the market. Indeed, most cryptocurrencies have been created by copy-pasting the open source code of bitcoin using a cryptocurrency generator such as http://build-a-co.in, mostly for pedagogical or branding purposes—and occasionally to lure naïve users into Ponzi schemes. Cryptocurrencies belonging to the latter group, and whose existence is typically short-lived, are not to be confused with the serious attempts at introducing value-creating innovations. Just as an empirical study of firm performance would not sample “shell” corporations—which are not designed for productive purposes—the present study does not consider “shell” cryptocurrencies—those currencies not backed by a team of developers aiming at creating improvements over existing alternatives [3].

For the purpose of this study, we focus on five cryptocurrencies whose major innovations were widely recognized by the community, and whose code has been audited and verified by multiple independent third parties. We decided to include bitcoin (BTC) since it represents the benchmark against which the value of other cryptocurrencies can be assessed. We then picked one cryptocurrency from each major wave of cryptocurrency creation. From the second wave of cryptocurrencies, which began in 2011, we chose to include Litecoin—the second cryptocurrency ever introduced. Litecoin (LTC) represents an improvement over BTC in terms of cybersecurity and processing speed. The third wave of cryptocurrencies, introduced in 2012, relied on a different mechanism to maintain network integrity, namely “proof-of-stake.” Peercoin (PPC) represents a prototypical example of a third-generation cryptocurrency, and we included it in our sample because it combines traditional proof-of-work (à la bitcoin) with the novelty of a proof-of-stake algorithm. The fourth wave of cryptocurrencies, heralded in 2013, sought to create value outside the realm of peer-to-peer payments. Ripple (XRP) represents an interesting case in point, with a team of developers managed by a for-profit organization called Ripple Labs, and a verification process that does not rely on mining to achieve consensus. The fifth wave, which started in 2014, consisted of cryptocurrencies seeking to combine advantages introduced in previous waves (e.g., instantaneous processing, use cases beyond payments) without compromising on openness and public auditability. Stellar (STR), created in August 2014, illustrates this endeavor well. Table 1 below summarizes basic information about the five cryptocurrencies examined in this study, which together account for more than 90% of the total market capitalization of all cryptocurrencies tradeable online as of 27 September 2015 [13].

**Table 1:** Five cryptocurrencies.

	Created	Main stated purpose	Technological features	Stated advantages compared with bitcoin	Maximum supply	Market capitalization (and rank)
**Bitcoin (BTC)**	03-Jan-09	Payment system	decentralized; mined using proof-of-work; SHA-256 hashing; block every 10 minutes		21 million	10 Aug 2014: $7.7bn (#1); 4 Jan 2015: $3.8bn (#1); 5 Jul 2015: $3.7bn (#1)
**Litecoin (LTC)**	07-Oct-11	Payment system	decentralized; mined using proof-of-work; Scrypt hashing; block every 2.5 minutes	faster verification for transactions; more resistant to double-spending attacks	84 million	10 Aug 2014: $216m (#2); 4 Jan 2015: $75m (#3); 5 Jul 2015: $167m (#3)
**Peercoin (PPC)**	12-Aug-12	Payment system	mostly decentralized; mined using proof-of-work and minted using proof-of-stake (1% annual rate); SHA-256 hashing; block every 10 minutes	energy efficiency makes it more scalable; proof-of-stake increases the cost of monopolizing the mining process and of launching "51% attacks"	grows long term at a 1% annual inflation rate	10 Aug 2014: $21m (#6); 4 Jan 2015: $11m (#10); 5 Jul 2015: $11m (#9)
**Ripple (XRP)**	01-Jul-13	Currency exchange, settlement, remittance	mostly decentralized; 100bn XRP pre-mined; ledger updated almost instantaneously; consensus based on Byzantine agreement with "starter" membership list	security, real-time money transfers, efficient international settlement	100 billion	10 Aug 2014: $43m (#3); 4 Jan 2015: 657m (#2); 5 Jul 2015: $334m (#2)
**Stellar (STR)**	04-Aug-14	Financial accessibility: exchange, settlement, remittance (unlike Ripple Labs, the Stellar Foundation is a non-profit)	decentralized; federated Byzantine agreement to achieve consensus; 100bn STR pre-mined; ledger updated almost instantaneously	adds low latency, flexible trust and asymptotic security to decentralization	100 billion	10 Aug 2014: $1.6m (#30); 4 Jan 2015: $16m (#9); 5 Jul 2015: $16m (#7)

### Observation Period

Our analyses of the five cryptocurrencies began in September 2014, shortly after the introduction of Stellar on popular online exchanges, and ended one year later, in August 2015. Since all these cryptocurrencies were still in existence in August 2015, we thus obtained balanced panel data, which allowed us to control for unobserved characteristics of each cryptocurrency and minimize the noise created by cross-panel heterogeneity. Data for some of the key variables were available only weekly, so we decided to aggregate all other variables at the week level to obtain a rich explanation of the drivers of cryptocurrency returns, and mitigate the noise created by relying on daily observations [14]. Our dataset includes 255 observations, but due to some variables being lagged in our models, we ran all of our analyses on 250 observations (i.e. the first observation, for each of the five cryptocurrencies, is only used as a lag).

### Dependent Variable and Model Overview

We decided to predict weekly cryptocurrency returns instead of price because finance theory rationalizes asset return as a reward for investors. “Finance contains many examples of theories implying that expected returns should be monotonically decreasing or monotonically increasing in securities’ risk or liquidity characteristics”[15]. An equally important reason for modelling return is its desirable statistical property, i.e. stationarity. In contrast, the price time series may not be stationary, which may result in spurious correlations [16, 17]. Indeed, a joint Im–Pesaran–Shin (IPS) test for panel data led us to conclude that we cannot reject the non-stationarity hypothesis for price time series (this result holds even after removing the time trend). Table 2 compares the stationarity test results for price and returns. IPS is the preferred test here because of sample size, and because it allows the time dimension dynamics of each panel, which drives non-stationarity, to vary.

**Table 2:** IPS Stationarity Tests (null hypothesis: All panels are non-stationary [joint]; The panel is non-stationary [panel by panel]).

Test Type	Remove Time Trend	Price	Returns
Joint	No	Fail to Reject	Strongly Reject
Joint	Yes	Fail to Reject	Strongly Reject
Panel by panel	No	Fail to reject for 4 out of 5 panels	Strongly reject for all panels
Panel by panel	Yes	Fail to reject for all panels	Strongly reject for all panels

For these reasons, our dependent variable, *weekly returns*, is computed as *[Price*_*t+1*_ *–Price*_*t*_*]/ Price*_*t*_. In the next section, we model *weekly returns* as a linear combination of various supply- and demand-side variables. We are especially interested in understanding which aspects of demand-side factors affect weekly returns, in particular, whether the “buzz factor” (as captured by indicators of *public interest* and *negative publicity*) and cryptocurrencies’ innovation potential (as captured by multiple indicators of *technological development*) attract or detract investors. Our models control for market *liquidity* and (unexpected) *supply growth*, for time-invariant unobserved heterogeneity (e.g., founders’ reputation, reliability of hashing algorithm) using cryptocurrency fixed effects, and for time-varying unobserved variables (e.g., stock market returns, regulatory environment) using a weekly time trend. Details on data, measures, and estimation method follow in the next section. To enhance causal inference, we lagged all our predictors except *liquidity*, which by design should have a contemporaneous effect on demand and returns. The basic model is specified as follows:

$$r_{i,t}=\alpha +\sum_{j}\beta_{j}x_{ij,t-1}+c_{i}+w_{t}+\varepsilon_{i,t}$$

Where *x*_*ij*,*t*−1_ is j^th^ predictor for i^th^ cryptocurrency, lagged by one period (except for *liquidity)*;

*c*_*i*_ is a cryptocurrency-specific fixed effect;

*w*_*t*_ is a weekly time trend;

*ε*_*i*,*t*_ is the unobserved error term for coin i in period t;

*α* is the intercept.

## Data and Measures

### Data Sources

We acquired data from CoinGecko.com, a leading source of information on cryptocurrencies. CoinGecko systematically collects data on various cryptocurrencies, including information on trading volume, price, market capitalization, and quantity in circulation. CoinGecko founders also developed and validated four longitudinal, multidimensional indicators to capture liquidity, developer activity, community support, and public interest [18]. For instance, the CoinGecko web application connects to the official application program interfaces (APIs) from Reddit, Facebook, Twitter, Github, and Bitbucket to continuously update the values taken by its indicators over time. For price and volume data, the API of a third-party price data provider is used. Market capitalization data were obtained from Coinmarketcap.com. Finally, we used the Factiva database to collect media coverage data on each cryptocurrency. All our data were aggregated at the week level and were collected for an entire year starting in September 2014.

### Measures

#### Dependent variable: weekly returns

**Fig 1:**
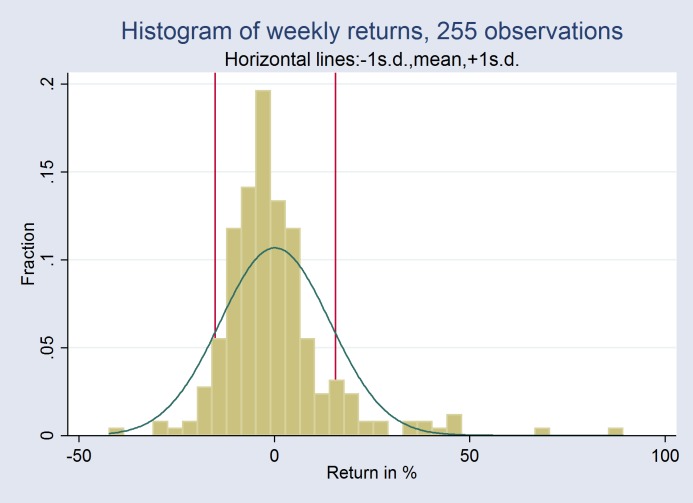
Distribution of Weekly Returns.

The price of each cryptocurrency was averaged across exchanges, and weighted using each exchange’s trading volume. We then computed *weekly returns* as [Pricet+1 –Price _t_]/ Price _t_. Fig 1 below plots the distribution of the dependent variable. A Jarque–Bera normality test failed to reject the normality hypothesis at a 5% level for skewness (p = 0.07) and kurtosis (p = 0.09) (taken separately), but a joint test yielded a p-value slightly below the 5% threshold (p = 0.04). Overall, the distribution of *weekly returns* was close to a normal distribution over our study period.

#### Independent variables

We captured what we call, in this study’s title, the “buzz factor” surrounding cryptocurrencies using two indicators: *public interest* and *negative publicity*. The latter is often assumed to decrease cryptocurrency prices by deterring future user adoption, leading to investor exit [19,20]. To capture *negative publicity*, we hired a graduate research assistant to count how many media articles were published each week that associated the name of a given cryptocurrency with some form of suspicious or fraudulent activity, using appropriate keyword searches in the Factiva database (i.e., “Bitcoin” AND (“fraud*” OR “hacked” OR “Ponzi” OR “scam” OR “theft”). For instance, using the latter search query, 36 unique articles were identified for the period 3–9 January, 2015. For Ripple and Stellar, we used a slightly more constraining search query to avoid capturing articles that have nothing to do with the two cryptocurrencies (i.e. unlike Bitcoin, Peercoin, and Litecoin, Ripple and Stellar are terms that can refer to something else than cryptocurrencies in the English language, so we used [“ripple” near7 (“bitcoin” OR “crypto*” OR “altcoin”)] AND (“fraud*” OR “hacked” OR “Ponzi” OR “scam” OR “theft”) to address this issue). One of the paper’s authors independently verified the number of extracted articles using the search queries on ten randomly selected periods of one day or one week, and the counts were either identical (eight times) or within five percent of the recorded value (two times). We logged the number of article counts (plus one) before inclusion in our models.

We measure *public interest* using the CoinGecko indicator computed as a weighted average of both the number of web search results obtained on Bing when searching a given cryptocurrency (e.g., “Litecoin”) and the official cryptocurrency website’s rank in the Alexa web traffic ranking (e.g., https://litecoin.info). Thus, *public interest* increases when more people look for information about the cryptocurrency (e.g., following word-of-mouth), and when more third-party websites (e.g., news websites, blogs, and corporate websites) mention the cryptocurrency. Thus, when more “buzz” surrounds a particular cryptocurrency, its *public interest* indicator will typically be much higher. CoinGecko calibrates public interest and other indicators by normalizing the raw value using the benchmark bitcoin value (both logged). For example,

$${Standardized\;web\;search\;score}_{litecoin}=\frac{ln(litecoin\;web\;searches)}{ln(bitoin\;web\;searches)}$$

We then captured cryptocurrencies’ innovation potential using eight indicators of *technological development* available in our CoinGecko data, including the number of unique collaborators contributing code to the project, the number of proposals merged in the core codebase, the number of issues raised by the community about the code and fixed by the developers, or the number of forks (for a full list of indicators, see Empirical Analyses below). In short, *technological development* captures progress made collaboratively to fix, update, and upgrade each cryptocurrency’s underlying software code, namely, its underlying technology. “Shell” cryptocurrencies typically have a score close to zero on this indicator after their launch, since no modification is made to the original code. Serious cryptocurrency projects such as those tracked in our study vary in the extent to which their technology is improved, and how sustained that effort is over time—two dimensions thoroughly captured by our measure. Note that CoinGecko weighted each of the eight indicators of *technological development* to reflect each indicator’s relative importance. In addition, more weight is given to indicators that would be more difficult to manipulate. Due to a confidentiality agreement with CoinGecko, we are unable to reveal the exact weightings, which they consider to be proprietary information.

#### Control variables

As mentioned earlier, the evolution of supply for each cryptocurrency comprises a large predictable component, which can easily be anticipated by market participants and thus should not affect price or returns. However, for mineable cryptocurrencies such as BTC, LTC, and PPC, the mining difficulty is adjusted periodically to maintain a target for block validation (e.g., 10 minutes for BTC) that is independent of the intensity of mining activity (e.g., new mining rigs entering or exiting the market). These adjustments go in hand in hand with temporary deviations from the average block validation time, which cause unexpected variations in supply in the short term. For non-mineable cryptocurrencies such as XRP and STR, the surprise element comes from the previously unannounced distribution of coins by the developers’ team, which can also have short-term effects on price and returns. To capture the unexpected variations in supply, we computed *supply growth* as [Supplyt+1 –Supply _t_]/ Supply _t_, using CoinGecko’s indicator of “Supply,” which measures the number of coins actually in circulation at any point in time.

We measured cryptocurrency *liquidity* using the CoinGecko score based on the trading volume for each cryptocurrency, as obtained from all the major online exchanges. We subsequently report a robustness test using an alternative measure of liquidity calculated based on Amihud’s formula [21]. Results remain the same. The more liquid a cryptocurrency, the easier for a participant to find a counterparty to trade with. Finally, we controlled for time-invariant unobserved heterogeneity using cryptocurrency fixed effects, and for time-varying unobserved variables using a *week* time trend.

## Empirical Analyses

### Model Estimation and Statistical Inference

Both random-effects (RE) and fixed-effects (FE) estimators rely on ordinary least-squares assumptions (e.g. equations must be correctly specified, each predictor must be strictly exogenous and linearly independent). When these conditions are met, theory states that FE estimation is unbiased and consistent. RE estimation requires an additional assumption: the group-level effect and the regressors must be independent to avoid omitted variable bias [22]. When this assumption is met, RE estimation is unbiased, consistent, and, because it utilized both the within- and between-group variation, efficient. Under this assumption, FE estimation is not efficient because it only utilizes the within-group variation. So, in our context, if the cryptocurrency-specific fixed effect is exogenous to other predictors, then we should opt for the RE estimator, and if not, for the FE estimator. Indeed, “the key consideration in choosing between an RE and an FE approach is whether *c*_*i*_ and *x*_*ij*_ are correlated” [22]. In our context, the cryptocurrency effect *c*_*i*_ captures unobservable properties such as the inherent managerial skills of cryptocurrency founders in nurturing a community, which could be correlated with past levels of *negative publicity* or *technological development*, and make the RE estimator biased.

In line with best practice, we used a Hausman test to assess which estimator is more suitable in our context [22]. Since the variance of the error terms may differ across cryptocurrencies, we resorted to the Sargan-Hansen (SH) statistic, which is robust to heteroscedasticity. The test indicated that the fixed effect (FE) estimator would be more appropriate in our context (p = 0.0001). As explained below, we estimate our fixed-effects panel least-squares regressions using a variety of standard errors, and our results remained stable across specifications.

If the dependent variable and a given regressor are unrelated but are both non-stationary, the regression analysis tends to produce a statistically significant relationship, i.e., a spurious regression. We applied a Dickey-Fuller Unit Root test to each panel and found that all *weekly returns* series were stationary (p < 0.001 for all panels). We rejected the non-stationarity hypothesis for *supply growth*, *negative publicity*, and *technological development* (p < 0.01 for all panels), but cannot rule it out for all panels for *liquidity* and *public interest*. However, panel cointegration tests for any combinations of the two variables that show up in the regressions provides evidence that they are cointegrated (p < 0.001), implying that their sources of non-stationarity cancel out. Therefore, regression results can be interpreted confidently as long as these variables are included simultaneously in the models.

We explicitly model the main effects of our primary predictors (*public interest*, *negative publicity*, and *technological development*) as linear relationships. We made this choice for three reasons. First, we have no theoretical reason to believe that a curvilinear relationship would be at work. This could have been the case, for instance, if a major exogenous shock had happened over our period of study, opening up a new era wherein the influence of one of our predictors would suddenly become much greater. Second, modeling relationships as non-linear can artificially inflate model fit and lead to the “overfitting” problem. Besides, scholars find that going beyond the linear case does not necessarily enhance the replication power of studies that predict hedge fund performance. Rather, selecting factors with a straightforward economic interpretation allows for a substantial out-of-sample performance improvement in replication quality, whatever the underlying form of the factor model [23]. In line with extant knowledge, we thus opted for a more conservative—and easier to interpret—linear test of our model. Third, we empirically tested for the presence of non-linear relationships by running our four main models after including, sequentially, squared terms for *public interest*, *negative publicity*, and *technological development*. Across the twelve models thus obtained, none of the coefficients on the squared terms approached a satisfactory level of statistical significance, with p-values ranging from 0.11 to 0.82 (mean = 0.51, S.D. = 0.26). To sum up, our choice to model relationships linearly is grounded in both theoretical considerations and empirical evidence.

### Summary Statistics, Correlations, and Regression Results

Table 3 below displays summary statistics and correlations.

**Table 3:** Summary Statistics and Correlations.

	Variable	Mean	S.D.	Min	Max	1	2	3	4	5	6	7
**1**	Weekly returns	0.17	14.04	-42.22	89.31	1						
**2**	Liquidity	0.13	0.05	0.03	0.27	0.02	1					
**3**	Supply growth (thousands)	27.7	179	0.01	2022	0.27*	-0.12	1				
**4**	Public interest	0.06	0.02	0.03	0.10	-0.04	0.59*	0.03	1			
**5**	Negative publicity	0.39	0.71	0.00	2.80	-0.03	0.69*	-0.09	0.86*	1		
**6**	Technological development	0.20	0.04	0.10	0.31	0.03	0.76*	0.01	0.65*	0.59*	1	
**7**	Alternative liquidity (Amihud)	2.26	3.39	0.00	18.11	-0.12	-0.20*	-0.08	-0.19*	-0.02	-0.44*	1
**8**	Alternative interest (community)	0.14	0.05	0.10	0.31	-0.04	0.84*	-0.11	0.84*	0.92*	0.65*	-0.01

* p-values < 0.05.

The variance inflation factor (VIF) is used as an indicator of multicollinearity. A VIF of 1 indicates no correlation among the *k*^*th*^ predictor and the remaining predictors. VIF values below 4 are considered very safe in terms of results interpretation, whereas values above 10 are considered problematic [24]. In our main model (i.e., model 6 in Table 4 below), the mean VIF is 3.37, and the maximum VIF is below 6. The next section reports a robustness test assessing the impact of this higher value, and we conclude that multicollinearity is not an issue in our results.

**Table 4:** Regression Results.

Model^1^	(1)	(2)	(3)	(4)	(5)	(6)
	Huber-White	Huber-White	Huber-White	Newey-West	2-way clustered	Driscoll-Kraay
Liquidity	1.99***	2.00***	2.13***	2.13**	2.13***	2.13**
	(0.568)	(0.637)	(0.625)	(0.870)	(0.679)	(0.915)
Supply growth since (t-1)	0.10**	0.18*	0.20**	0.20**	0.20***	0.20***
	(0.044)	(0.091)	(0.077)	(0.086)	(0.045)	(0.042)
Public Interest_(t-1)_		-3.14	-4.94***	-4.94***	-4.94^2^	-4.94***
		(2.127)	(1.327)	(1.782)	(n/a)	(0.765)
Negative Publicity_(t-1)_		0.049	0.052	0.052	0.052	0.052
		(0.069)	(0.069)	(0.059)	(0.058)	(0.050)
Technological Development_(t-1)_			1.96***	1.96***	1.96***	1.96**
			(0.427)	(0.660)	(0.656)	(0.746)
Cryptocurrency fixed effects	Incl.	Incl.	Incl.	Incl.	Incl.	Incl.
Week trend	-0.00064	-0.0015	-0.0013	-0.0013	-0.0013	-0.0013
	(0.001)	(0.001)	(0.001)	(0.001)	(0.001)	(0.001)
Constant	-0.18**	0.069	-0.25	-0.30		-0.25
	(0.076)	(0.183)	(0.178)	(0.233)		(0.215)
*N*	250	250	250	250	250	250
Within- or adjusted-*R*^2^	0.04	0.06	0.09	0.06	0.09	0.09

^1^ To mitigate a potential endogeneity issue caused by simultaneous causality, models 1–3 instrument liquidity, the only variable not lagged by one period, using all the regressors.

^2^ The estimated variance-covariance matrix is not positive semi-definite for this coefficient, so the standard error cannot be estimated. This outcome happens occasionally with two-way robust estimation.

S.E. in parentheses.

* p<0.10.

** p<0.05.

*** p<0.01.

Models 1 to 3 in Table 4 below are estimated using Huber-White standard errors [25, 26], robust to heteroscedasticity. Model 4 computes Newey-West standard errors [27], robust to heteroscedasticity and autocorrelation (up to five lags). Model 5 computes two-way clustered standard errors [28], robust to arbitrary correlation both within panels and within time period. Model 6 computes Driscoll and Kraay standard errors [29], robust to heteroscedasticity, autocorrelation, and non-independence across panels. Given the structure of our data, Driscoll and Kraay standard errors are the preferred specification, as well as the one resulting in the highest R^2^ statistic. The latter also yields the most conservative standard error estimate for our primary variable of interest, *technological development* (i.e., compare model 6 with models 3, 4, 5 below).

Model 1 includes control variables, fixed effects, and the time trend. In model 2, we added the “buzz” indicators, namely *public interest* and *negative publicity*. Model 3 represents the full model, including *technological development*, estimated using Huber-White standard errors. Models 4, 5, and 6 replicate this full model with alternative standard error computations to test the robustness of our findings.

### Interpretation of the Findings

Looking across models, we find that *technological development* is positively and significantly (p< 0.001) associated with weekly returns. Specifically, we calculate that a one standard deviation (s.d.) increase in *technological development* corresponds to a 9% increase in returns (i.e., 0.046 × 1.96 = 0.09016). For the standardized log score to increase by one s.d., all components need to increase by the percentages listed in Table 5 below (the percentages differ because each component enters into the score not directly, but only after being standardized by a different denominator, i.e., the BTC counterpart). Improving one aspect without affecting the others is unrealistic, due to their correlations. So, it is more reasonable discuss the consequence of a simultaneous improvement in all components of technological development.

**Table 5:** Association between returns and indicators of technology & public interest.

**For each indicator, % increase leading to 1 s.d. increase in Technological Development**	
Github Stars	17.8%
Github Subscriber	13.8%
Github Total Issue	15.3%
Github Percentage of Closed Issues over Total Issues (different scale, indicator measured as percentage)	0.36%
Forks	17.1%
Average number of Commits in Last 4 Weeks	7.6%
Merged Pull Requests	16.0%
Unique Contributors	11.3%
***Resulting change in weekly return***	***9*.*0%***
**For each indicator, % increase leading to 1 s.d. increase in Public Interest**	
Bing Search	15.2%
Alexa Ranking	8.8%
***Resulting change in weekly return***	***-10*.*3%***

Our next interesting finding is the negative association between *public interest* and cryptocurrency returns. While it has often been assumed that greater visibility in the public sphere, including in the media, would create a buzz affecting cryptocurrency prices positively, our models do not support this idea. To the contrary, we find that a one s.d. increase in *public interest* (0.021) corresponds to a 10% decrease in returns (i.e., 0.021 × 4.94 = 0.10374).

Table 5 below reports the percentage increase required in each component to achieve a one s.d. increase in *technological development* and in *public interest*. The bottom row in each section reports the resulting impact on *weekly returns*.

Surprisingly, *negative publicity* is not significantly associated with returns. Put simply, we do not find any evidence that bad press affects price. However, given that *negative publicity* is highly correlated with *public interest*, we reran the full model without *negative publicity* to see whether the channel through which the latter affects returns is related to public interest. If so, this relationship could explain the negative coefficient on *public interest*. Besides, *negative publicity* has the highest VIF in our data (5.91), so running the model without it tests if our estimates are affected by multicollinearity. As shown in Table 6‘s model 7 below, the effect of *public interest* remains substantially the same with or without *negative publicity*, which indicates that the two are largely independent. Other coefficients remain stable. As expected, in model 7, the mean VIF has substantially decreased from 3.37 to 1.89, and the highest VIF is now 2.76 (for *technological development*). This confirms that multicollinearity was not an issue in our initial estimates.

**Table 6:** Robustness tests.

	(7)	(8)	(9)		(10)
	without *negative publicity*	alternative *liquidity* measure	alternative *interest* measure		GARCH-in-mean
Liquidity	2.01**		2.36**	*μ*	-0.044**
	(0.887)		(1.087)	(mean of return)	(0.014)
Liquidity—Amihud		0.013***		*ω*	0.003***
		(0.004)		(mean of Vol)	(0.001)
Supply Growth	0.19***	0.17***	0.08**	*α*	0.41***
	(0.045)	(0.045)	(0.036)	(return on Vol)	(0.124)
Public interest_(t-1)_	-4.66***	-5.56***		*β*	0.25**
	(0.829)	(1.076)		(Lag Vol on Vol)	(0.084)
Community interest_(t-1)_			-2.31	*λ*	5.87***
			(2.85)	(Vol on Mean)	(1.61)
Negative publicity_(t-1)_		0.038	0.037		
		(0.049)	(0.052)		
Technological development_(t-1)_	1.95**	1.62**	1.70*		
	(0.746)	(0.699)	(0.934)		
Cryptocurrency fixed effects	Incl.	Incl.	Incl.		
Week Trend	-0.0011	-0.0017	-0.0003		
	(0.001)	(0.001)	(0.001)		
Constant	-0.25	0.19	-0.29		
	(0.216)	(0.191)	(0.279)		
Observations	250	250	250		126
Adjusted *R*^2^	0.09	0.07	0.07	Log likelihood	135.9

S.E. in parentheses.

* p<0.10.

** p<0.05.

*** p<0.01.

The positive and significant coefficient observed across models for *supply growth* warrants discussion. In the commonly accepted Quantity Theory of Money [12], applicable to fiat currencies, an increase in supply leads, ceteris paribus, to a decrease in price. Note that this effect is also consistent with the commonsense understanding of supply and demand mechanisms—more supply decreases price, and more demand increases price. In our models, we find that more supply will increase price (and returns), which points to cryptocurrencies behaving differently from fiat currencies. We see at least two mechanisms that set cryptocurrencies apart and may result in the observed positive association between supply and returns. First, a short-term increase in supply may incite existing cryptocurrency holders to reinforce their position aggressively, and such display of confidence may, in turn, induce outsiders without prior awareness of cryptocurrencies to participate and buy coins. Second, an increased supply in the short term is likely the result of a spike in mining intensity, which could be interpreted as a signal of the cryptocurrency’s increasing potential to become a widely used medium of exchange. In both situations, the unexpected supply growth would result in a rightward shift of the demand curve, thereby driving up returns. The positive coefficient that we find on *supply growth* implies that these two demand-side mechanisms dominate the supply-side effect advanced in the Quantity Theory of Money; thus, the latter becomes insufficient to explain the behavior of cryptocurrencies. In other words, cryptocurrencies are not similar enough to traditional fiat currencies to obey the same rules.

Finally, in line with extant theory on financial assets, we find that *liquidity* is positively and significantly (p < 0.05) associated with returns. Indeed, a large sale order of a liquid asset could be easily executed at short notice without putting too much downward pressure on the market price. If only a few shares are traded every day, sellers need to keep lowering the price until they find enough buyers to take over the amount of shares they want to trade, a phenomenon known as *price slippage* [30]. Apart from price slippage, there is also an indirect opportunity cost for asset holding because people value money over other types of stores of values (a liquidity preference theory that originated from Keynes) [31].

## Supplementary Analyses

### Alternative Measures

#### Liquidity

A widely used measure of liquidity in the financial literature is the one proposed by Amihud [21]. The underlying idea is that as trading volume decreases, the corresponding asset becomes more difficult to trade in the short term, resulting in illiquidity. A symptom of illiquidity is the notable price change for a given amount of trade executed (the *price slippage* phenomenon mentioned in previous section). We computed an alternative measure of liquidity following Amihud’s formula:

$$Illiquidity=\frac{1}{D}\sum_{t}\left|R_{t}\right|V_{t}$$

where |*R*_*t*_| is the absolute value of daily returns, and *V*_*t*_ is the respective daily volume in dollars. This ratio reflects the daily price impact of the trading flow. We compute the weekly illiquidity as a seven-day average of this ratio, i.e., D = 7.

We then multiplied this illiquidity measure by −1 to obtain a measure of liquidity directly comparable with our initial measure. Model 8 in Table 6 shows that the coefficient on *Amihud’s liquidity* remains positive and significant (p < 0.01). Note, however, that our initial measure of *liquidity* explained our data better, as visible in the higher R^2^ statistic in model 6 compared to model 8.

#### Public interest

A surge in *public interest* is negatively associated with returns. To assess the robustness of this finding, we ran a supplementary analysis using an alternative indicator, which we call *community interest*. This alternative measure consists of a weighted average of six CoinGecko indicators that capture activity in social media channels: the number of Reddit subscribers, the number of active Reddit users, new Reddit posts in the previous 48 hours, new Reddit comments in new posts, the number of Likes on the coin’s official Facebook page, and the number of followers on the coin’s official Twitter account.

While *public interest* captures interest from an audience of outsiders (e.g., prospective cryptocurrency users), *community interest* focuses more on an audience of community insiders (e.g., existing cryptocurrency users). Model 9 in Table 6 shows that the coefficient on *community interest* is negative (though not significant), in line with our main measure of *public interest*. Other coefficients remain stable.

We wanted to further validate our use of CoinGecko’s *public interest* indicators and of our own *negative publicity* variable to capture the “buzz factor” surrounding cryptocurrencies. To that end, we collected from the Factiva database the total weekly number of articles mentioning each cryptocurrency—arguably a good measure of *media visibility* (i.e. Factiva combines more than 36,000 media sources). Table 7 below shows how our two primary indicators of the “buzz factor”, *public interest* and *negative publicity*, correlate with such *media visibility*, as well as with the alternative indicator of interest we termed *community interest*. Pairwise correlations range between 0.86 and 0.93, indicating high levels of internal validity.

**Table 7:** Correlations between various indicators of the “buzz factor” surrounding cryptocurrencies.

	Variable	Mean	S.D.	Min	Max	1	2	3
**1**	Public interest	0.06	0.02	0.03	0.10	1		
**2**	Negative publicity	0.39	0.71	0.00	2.80	0.86	1	
**3**	Media visibility (robustness test)	0.86	1.34	0	4.42	0.88	0.96	1
**4**	Community interest (alternative measure)	0.14	0.05	0.10	0.31	0.84	0.92	0.93

### Investor Expectations and Volatility

To further understand why the “buzz factor” is negatively, rather than positively, associated with returns, we go beyond modeling the average *weekly returns* and seek to understand the drivers of their variance, or “volatility.” Our rationale is the following: “buzz” could affect the expected uncertainty regarding future returns, that is, their volatility. More specifically, a sudden increase in the “buzz” surrounding a cryptocurrency could be interpreted as a signal of increasing volatility. If market participants are risk-averse, given the same expected mean returns, they would be less willing to hold the cryptocurrency if future volatility increases, which would drive prices down and affect returns negatively. This effect would become evident shortly after the surge in “buzz.” To assess the plausibility of this scenario, we model the relationship between average returns and their variance using a Generalized Autoregressive Conditional Heteroscedasticity (GARCH) model [32].

Unlike returns (*r*), volatility (*σ*) is unobservable. GARCH is a simple volatility model that accommodates time-varying variances. GARCH models are popular in finance because they capture a common feature embedded in financial returns—the long-run distribution of the returns exhibiting non-normality, i.e., fat tails and skewness. This consequence is an outcome of time-varying variances (heteroscedasticity), which non-dynamic linear models with Gaussian assumptions fail to capture. Put simply, GARCH models capture the fact that errors can be unevenly distributed over time, with bursts of positive or negative errors occurring over extended periods. Here, we are exploring the possibility that bursts of positive or negative errors could be associated with sudden variations of public interest around particular cryptocurrencies.

In the following, we use a GARCH-in-the-mean model [33], which allows variance to affect the mean directly through the term *λσ*_*t*+1_.

$$r_{t+1}=\mu_{t+1}+\sigma_{t+1}z_{t+1}$$

$$\sigma_{t+1}^{2}=\omega +\alpha r_{t}^{2}+\beta \sigma_{t}^{2}$$

$$\mu_{t+1}={\mu +\lambda \sigma_{t+1}\lambda \sigma }_{t+1}$$

The variance *σ*_*t*+1_ is assumed to be centered around an unconditional mean ω and positively correlated with *σ*_*t*_. The mean of returns μ_*t*+1_ changes over time. Investors expect the risk premium *λσ*_*t*+1_ to increase as volatility goes up. The uncertain component of the returns *σ*_*t*+1_*z*_*t*+1_ is driven by the normally distributed noise term *z*_*t*+1_. A comparison between the basic GARCH model and the GARCH-in-the-mean model confirms that the latter fits our data better (i.e., we obtain smaller values for log-likelihood, Akaike information criterion, and Bayes information criterion). Model 10 in Table 6 above reports the main statistics for the GARCH-in-the-mean model. This model is used to fit the 126 *weekly returns* data available for BTC. We also estimated GARCH-in-the-mean models for the four other cryptocurrencies, and the results are consistent with the BTC model.

The positive and significant (p < 0.000) coefficient *λ* is consistent with investors’ aversion for volatility, that is, they are satisfied with a lower risk premium (in the form of lower mean returns) accompanied by lower volatility. Bursts of *public interest* could lead people to expect lower future volatility *σ*_*t*+1_ and thus lower returns *r*_*t*+1_. This expectation is reasonable because a surge in buzz can feed speculation, leading to a price correction in subsequent periods. Indeed bitcoin developer Mike Hearn notes that media hype sometimes “accelerates until it turns into a pure speculative bubble which then pops, leaving the price down from the peak” [9].

## Discussion

Since 2009, audiences such as journalists, regulators, and business observers have struggled to categorize entities such as bitcoin and litecoin. Are these entities more similar to “money,” “virtual currencies,” “assets,” or “commodities”? Scholars have also tackled this difficult challenge. For instance, Bjerg [34] argues that “bitcoin is commodity money without gold, fiat money without a state, and credit money without debt.” In quantitative studies of bitcoin prices, the baseline assumption has often been that bitcoin is somewhere between a financial asset and a currency—so theories about asset and currency pricing are both relevant to examine its properties. This assumption, reasonable at first sight, leads to expectations that, as bitcoin supply increases, it price should decrease (i.e., this effect is what we expect from a currency, as per the Quantity Theory of Money).

Cryptocurrency can be seen either as currency or as commodity. Although these two share several economic properties, there are key differences. While the former is a representation of value, the latter carries value. And, while “bitcoin represents an innovation that does not easily fit into a superordinate category” [35], our study departs theoretically from prior accounts by acknowledging upfront that cryptocurrency is technology, and consequently is underpinned by a potential for innovation that can have value on its own. We test this idea by using unique data capturing various dimensions of *technological development*, which we find to be positively and significantly associated with weekly returns. This implies that treating cryptocurrency as currency is insufficient to understand the phenomenon. Strictly speaking, this study shows that cryptocurrency is neither currency nor commodity. Thus, our findings should lead scholars to treat cryptocurrencies as technology platforms, and not just as financial or monetary instruments [36]. Conceiving of cryptocurrency as technology implies that it can have various use cases and applications (e.g., payments, smart contracts, record keeping), each of which can create a certain amount of value (e.g., by capturing market share in a corresponding industry, or by creating a new industry altogether). Besides, a new cryptocurrency may look more appealing than its older competitors at the time of introduction, but if it is not backed by a solid team of developers who continually improve its underlying software, over time it will be unable to maintain its initial technological advantage (even less so if its software code is open source and can be easily copied by pre-existing competitors, which is typically the case). These findings complement the claim that cryptocurrency could be seen as “synthetic commodity money”, which “resembles fiat money in having no nonmonetary value [but also] resembles commodity money in being not just contingently but absolutely scarce” [37]. Therefore, future research should account for the technological dimension of cryptocurrency explicitly and dynamically (i.e., software upgrades can happen on a weekly basis).

Our study also departs empirically from prior work in two important dimensions. First, we look at a representative panel of cryptocurrencies, and not just bitcoin [3, 4, 5, 9, 10, 38, 39, 40, 41, 42, 43, 44]. Second, we look at a recent time period, no longer characterized by the massive volatility and price bubbles of the early bitcoin years (i.e., 2009 to 2013). Our results should thus be interpreted with this recent time period in mind, and may not generalize to other time periods [45]. As well, we anticipate that the returns of “shell” cryptocurrencies (e.g., HotCoin), which do not seek to introduce technological innovation, might be explained by a different set of factors—including illegal attempts at influencing their price. Notwithstanding these boundary conditions, our study suggests a clear answer to our initial question regarding what explains cryptocurrency returns—in short: technology truly matters.
