# Correction: Buzz Factor or Innovation Potential: What Explains Cryptocurrencies' Returns?

**DOI:** 10.1371/journal.pone.0177659

**Published:** 2017-05-10

**Authors:** Sha Wang, Jean-Philippe Vergne

There is an error in the equation under the subheading “Liquidity” in the Supplementary Analyses section. The correct equation is:

$$Illiquidity=\frac{1}{D}\sum_{t}|R_{t}|/V_{t}$$

There is an error in the third equation under the subheading “Investor Expectations and Volatility” in the Supplementary Analyses section. The correct equation is:

$$\mu_{t+1}=\mu +\lambda \sigma_{t+1}$$
